# Prophylactic mesh placement of permanent stomas at index operation for colorectal cancer

**DOI:** 10.1308/003588412X13373405386493

**Published:** 2012-05

**Authors:** NT Ventham, RR Brady, RG Stewart, BM Ward, C Graham, S Yalamarthi, M Jones, T Daniel

**Affiliations:** ^1^NHS Fife,UK; ^2^University of Edinburgh,UK

**Keywords:** Parastomal hernia, Paracolostomy hernia, Prophylactic mesh, Computed tomography

## Abstract

**INTRODUCTION:**

Parastomal herniation occurs in 30–50% of colostomy formations. The aim of this study was to radiologically evaluate the mechanical defects at stoma sites in patients who had previously undergone a permanent colostomy with or without mesh at the index operation for colorectal cancer.

**METHODS:**

A study was performed of all colorectal cancer patients (*n*=41) having an end colostomy between 2002 and 2010, with or without Prolene® mesh plication, with blinded evaluation of the annual follow-up staging computed tomography (CT) for stomal characteristics. The presence of parastomal hernias, volume, dimensions, grade of the parastomal hernia and abdominal wall defect size were measured by two independent radiologists, and compared with demographic and operative variables.

**RESULTS:**

In those patients with radiological evidence of a parastomal hernia, Prolene® mesh plication significantly reduced the incidence of bowel containing parastomal hernias at one year following the procedure (*p*<0.05) and also reduced the diameter of the abdominal wall defect (*p*=0.006).

**CONCLUSIONS:**

Prophylactic mesh placement at the time of the index procedure reduces the diameter of abdominal wall aperture and the incidence of parastomal hernias containing bowel. Future studies should use both objective radiological as well as clinical endpoints when assessing parastomal hernia development with and without prophylactic mesh.

A parastomal hernia is an incisional hernia related to an abdominal wall stoma.^1^ While stomal complications are common, parastomal hernias are the most common complication with an incidence of 30–50%.^2–4^ Although most cases of parastomal hernia are asymptomatic, presenting symptoms range from poorly fitting stoma devices and unsatisfactory cosmesis to more severe presentations such as strangulation and obstruction.^2^ While the indications for repair vary between units, the absolute indications include strangulation and obstruction, with intermittent obstructive symptoms, pain, poor cosmesis and skin excoriation from poorly fitting devices being relative indications.^2^ Some studies report repair rates of up to 30%, and repair has an associated morbidity and high recurrence rate.^2–4^ More recently, there has been a focus on preventing rather treating parastomal hernias.^3^

Herniation following stoma formation has a higher incidence after colostomy compared with ileostomy.^2,5,6^ Other risk factors include obesity, increasing age, corticosteroid use, nutritional deficiencies and chronic respiratory disease.^3^ Technical aspects such as aperture size in the abdominal wall through which the stoma passes are also relevent.^3^

In addition to clinical examination, radiological investigations have been used to identify and classify parastomal hernias.^7–11^ These include supine computed tomography (CT) and dynamic CT with Valsalva manoeuvres or alternative positions (eg sitting, prone). While there has been discordance between the clincial and radiological correlation of parastomal hernias,^10,11^ radiological diagnosis has been demonstrated to be superior to clinical examination, which has been shown to underdiagnose the presence of parastomal hernias.^2,7–9^

Placement of lightweight polypropelene mesh at the index operation has been shown to reduce the rate of parastomal hernias. Mesh placement may be in the sublay (preperitoneal/submuscular),^12–15^ intraperitoneal^16^ or onlay positions.^17^ A meta-analysis published in 2010,^4^ including three randomised controlled trials (RCTs),^12,13,18^ has shown this intervention is effective in preventing parastomal hernias: 55.2% of patients developed parastomal hernia in the non-intervention group versus 15.4% with a mesh placement (*p*<0.0001). Mesh placement is generally regarded as safe with few (<5%) complications reported.^4^

The major flaw with the current randomised trials is the lack of predefined endpoints regarding parastomal hernias. Other trials with clinical and ultrasonographic assessment had smaller numbers of patients.^12^ Serra-Aracil *et al* used a combination of clinical and CT endpoints in colorectal cancer patients but the data failed to reach statistical significance when comparing mesh with no mesh in preventing parastomal hernias on CT.^13^ In two of the RCTs there were varied indications for stoma formation^12,18^ and one trial compared herniation in ileostomy and colostomies.^12^

The aim of this study was to radiologically evaluate the mechanical defects at the stoma site in patients who underwent a permanent colostomy with or without mesh at the index operation for colorectal cancer.

## Methods

The patient cohort was identified from a prospectively gathered cancer database between the years of 2003 and 2010 at Queen Margaret Hospital, NHS Fife. Strict inclusion and exclusion criteria were instituted to provide similar cohorts for comparison. Patients were included if they had colorectal cancer resected electively, with formation of a colostomy at the index procedure by one of two colorectal surgeons (TD, SY). Patients were excluded from the analysis if they had no annual surveillence CT, including those who died within the first year following surgery, those with benign disease and those whose CT was performed later than two years following surgery. Patients were also excluded if they had their operation in an emergency setting or if there were missing/non-accessible notes or radiology results.

Patient demographic details were recorded along with peri-operative factors including pre-operative co-morbidities. Post-operative stoma specific complications were also recorded.

### Operative methodology

All patients underwent an open Hartmann’s operation or abdominoperineal excision of the rectum (APER) by one of two senior surgeons (who were the primary operator in all cases). All end colostomies were fashioned using a standard technique throughout the study period, using a trephine technique with the end stoma brought out through the rectus abdominis muscle. The use of prophylactic mesh placement has been developed in our unit during the study period by two of the senior authors (TD, SY) and has now become stardard practice for elective colostomy formation.

All meshes were placed in the sublay position in line with previous reports.^12,13,18^ A 15cm × 9cm lightweight part polypropelene/part absorbable Prolene® mesh (Ethicon, Somerville, NJ, US) was placed between the rectus muscle and posterior rectus sheath during stoma formation and the mesh was trimmed to size (aiming for a coverage of approximately 3cm around the stoma). The closed end of the stoma was passed through a circular defect in the mesh, the size of which is dictated by the size of the stoma and mesentry. The mesh was not fixed (relying on the inflammatory and subsequent fibrotic reaction to fix the mesh).^12,15^

### Radiological follow-up

As part of the post-operative cancer surveillance, patients proceeded to a routine first annual follow-up CT scan. Analysis of this imaging was undertaken by two independent radiologists who were blinded to the placement of a mesh (meshes were not identifiable on annual CT). Multiplanar reformats were performed using Vitrea® workstations (Toshiba, Crawley, UK) to develop reproducible orthogonal views of the stomas and anterior wall defect.

Radiological measurements included the dimensions of:

> the abdominal wall stomal defect (volume, transverse diameter)
> the stoma (volume, transverse and anteroposterior measurements)
> the parastomal hernia sac (volume, transverse and anteroposterior measurements)

The hernia contents were also recorded (omentum, small bowel, large bowel separate to stoma, stoma bowel itself). The parastomal hernias were graded radiologically using the scale developed by Moreno-Matias *et al*.^9,13^ This graded hernias as:

> Ia: hernia sac containing bowel forming stoma (sac diameter <5cm)
> Ib: hernia sac containing bowel forming stoma (sac diameter >5cm)
> II: hernia sac containing bowel forming stoma with omentum
> III: hernia sac containing intestinal loop other than that forming stoma

Variables were recorded on a proforma and entered into an Excel® worksheet (Microsoft, Redmond, WA, US). Analysis was performed using Minitab® 15 (Minitab, Coventry, UK). In order to compare categorical data between groups, we used a test for comparison of proportions (where variable has two levels) or the chi-squared test (where variable has more than two levels). Results have been presented from Fisher’s exact test where appropriate owing to small counts. For continuous data, two-sample t-tests were used. Statistical significance was defined as *p*<0.05.

## Results

Between 2003 and 2010, 37 (54%) APER and 31 (46%) Hartmann’s operations were performed for rectal or rectosigmoid cancer by the two senior authors. Overall, 41 patients met both the inclusion criteria and had a one-year CT surveilence scan (mean time to CT 58 weeks) and 27 patients were excluded (4 CT scan >2 years, 4 non-rectosigmoid tumour, 2 emergency operation, 2 deaths within 1 year, 3 notes/12 CT results not available). Forty-one per cent (*n*=17) received a prophylactic mesh at the time of the index procedure.

Table 1 shows the comparative demographics of patients in each of the treatment groups (mesh vs non-mesh). There was no evidence of a statistically significant difference in age, sex, body mass index or pre-operative co-morbidities between the two groups. None of the patients had a pre-existing incisional hernia.

**Table 1:** Comparison of variables in intervention group versus non-intervention group

Demographics	Non-mesh (*n*=24)	Mesh (*n*=17)	Difference in means (95% CI)	*P*-value
Mean age (SD)	67.94 yrs (8.72 yrs)	68.58 yrs (10.29 yrs)	-0.64 (-6.68–5.40)	0.831
Mean body mass index (SD)	27.65kg/m^2^ (4.21kg/m^2^)	27.63kg/m^2^ (4.66kg/m^2^)	0.02 (-2.81–2.85)	0.988
Sex (female)	11 (65%)	14 (58%)	6.4 (-23.7–36.5)	0.678
Hartmann’s operation	7 (41%)	5 (21%)	20.3 (-8.1–48.8)	0.162*
Co-morbidities				
Respiratory	1 (6%)	6 (25%)	-19.1 (-39.7–1.5)	0.207*
Diabetes	3 (18%)	2 (8%)	8.3 (-11.9–30.5)	0.633*
Pre-operative radiotherapy	11 (65%)	13 (54%)	10.5 (-19.7–40.8)	0.539

SD = standard deviation; CI = confidence interval

* Fishers exact test

In the study population, stoma specific complication rates were very low with no patients experiencing stenosis, necrosis or reoperation. There was one patient (non-mesh) who developed stoma retraction and three patients with midline wound infection (2 mesh, 1 non-mesh). There were no cases of mesh erosion in the study period.

Table 2 provides the radiological findings and recorded details of the type and grade of hernia in the two treatment groups using the classification system developed by Moreno-Matias *et al*.^9^ Their study demonstrated that radiological grade II and III hernias were most commonly symptomatic and apparent on clinical examination whereas grade Ia and Ib hernias were often clinically unapparent and asymptomatic. We divided parastomal hernias into two groups for analysis: subclinical (none, grades Ia and Ib) and clinically relevant (grades II and III). In the mesh group, 35% (6/17) had a clinically relevant parastomal hernia compared with 54% (13/24) in the non-mesh group (difference in proportions 19%, 95% confidence interval [CI]: -11–49%, *p*=0.221). There were no grade III parastomal hernias in the mesh group compared with seven (29%) in the non-mesh group (difference in proportions 29%, 95% CI: 11–47%, *p*=0.029).

**Table 2:** Radiological findings in relation to grade and contents of parastomal hernia in treatment groups

		Non-mesh (*n*=24)	Mesh (*n*=17)
**Hernia contents**	No hernia	10 (42%)	7 (41%)
	Omentum	6 (25%)	6 (35%)
	Small or large bowel	7 (29%)	0 (0%)
	Bowel forming stoma only	1 (4%)	4 (24%)
**Hernia grade**	None	10 (42%)	7 (41%)
	Ia	0 (0%)	1 (6%)
	Ib	1 (4%)	3 (18%)
	II	6 (25%)	6 (35%)
	III	7 (29%)	0 (0%)

**Table 3:** Radiological findings in relation to physical dimensions of hernia sac and defect of parastomal hernia in treatment groups

		Non-mesh (*n*=24) Mean (SD)	Mesh (*n*=17) Mean (SD)	Difference in means (95% CI)	P-value
**Defect**	Volume (mm^3^)	50.56 (14.42)	41.99 (8.74)	8.6 (1.21–19.92)	**0.024**
	Transverse (mm)	27.90 (13.93)	18.61 (5.33)	9.29 (2.92–15.66)	**0.006**
**Stoma**	Volume (mm^3^)	20.85 (7.16)	18.58 (5.14)	2.28 (-1.61–6.17)	0.243
	Transverse (mm)	15.66 (8.00)	13.34 (4.31)	2.32 (-1.61–6.25)	0.239
**Hernia sac in those with hernia (14 non-mesh, 10 mesh)**	Volume (mm^3^)	63.89 (22.04)	56.72 12.71	7.17 (-7.65–22.00)	0.326
	Transverse (mm)	79.54 (28.63)	57.09 23.47	22.4 (0.3–44.6)	**0.047**
	Anteroposterior (mm)	43.62 (14.60)	37.67 8.80	5.95 (-4.01–15.92)	0.228

SD = standard deviation; CI = confidence interval

In those patients with a mesh, there was evidence of a smaller transverse abdominal wall defect by 9.29mm (95% CI: 2.92–15.66mm; *p*=0.006). In those with a parastomal hernia, patients with a mesh had a smaller volume and dimensions of hernia sac but this trend did not reach significance (volume: non-mesh 63.89mm^3^ vs mesh 56.72mm^3^, *p*=0.326; diameter: non-mesh 79.54mm vs mesh 57.09mm, *p*=0.047).

## Discussion

The parastomal hernia rate in this study in those patients without prophylaxic mesh was 54% and is similar to that reported in a systematic review from 2010 (55.2%).^4^ Our early experience in using prophylactic mesh resulted in a rate of parastomal hernias of 35%. While this is higher than the 15.2% published in the same meta-analysis above, our data reflect the detection of early hernia development by abdominal CT and may also be a reflection of the learning curve involved in instituting this technique.^4^

In those patients with a prophylactic mesh, the incidence of radiological grade III hernia was significantly lower (29% non-mesh vs 0% mesh, *p*=0.003). It has been demonstrated previously that grade III hernias are almost always symptomatic, with decreasing symptomatology associated with lesser hernia grades.^9^ For instance, grade II hernias are symptomatic 60% of the time while grade Ib hernia symptoms are reported in 40% of cases.

Although not all parastomal hernias require intervention, repair is more likely to be required for symptomatic hernias.^4^ In this case, mesh prophylaxis preventing symptomatic hernias would be beneficial. None of the parastomal hernias in the mesh group contained large or small bowel. Conversely, 46% of the parastomal hernias in those patients without a mesh contained large or small bowel. While the absence of bowel in parastomal hernias in those with a prophylactic mesh is an important early observation at one year following surgery, in the longer term, bowel loops may herniate into the sac. Analysing the radiological contents of parastomal hernias in those with a prophylactic mesh over a longer follow-up duration would be helpful in clarifying this.

This study attempted to describe the size of the stomal defect as well as the dimensions of the parastomal hernia sac if present. We confirmed that the transverse diameter defect in the abdominal wall through which the stoma passes was significantly smaller in those patients with a mesh. It has been suggested that a larger opening in the abdominal wall is associated with an increased risk of herniation.^3^ A smaller defect should therefore prevent prolapse of viscera.

In a descriptive study published in 2010, Pilgrim *et al* explained that abdominal wall aperture size is independently predictive of parastomal hernia formation.^19^ They stated that for every milimetre of aperture size the risk of parastomal herniation increased by 10% (*p*=0.005). This study demonstrates that prophylactic mesh significantly reduces abdominal wall aperture size and may therefore explain the lower rates of parastomal herniation in those with a prophylactic mesh. The described difference in aperture size associated with the placement of mesh is presumed to be due to the mesh itself as there were no other methodological differences in stoma formation. We also found that the transverse diameter and volume of the hernia sac in those with mesh tended to be smaller, a result that failed to reach significance.

Our findings confirm reports that mesh placement is safe. There was no statistically significant difference in stoma specific complications between the mesh and non-mesh groups. In the mesh group there was only one local wound infection and no peri-stomal complications. No patients required reoperation. There were no cases of stoma stenosis or mesh erosion in the mesh group during the study period. The lightweight meshes used may have contributed to the low levels of infection.^18^ The placement of sublay mesh may have contributed to the low local infective complications as the mesh is placed outside the peritoneal cavity.^18^ The bowel was opened to create the stoma only after it had passed through the mesh, thereby avoiding exposure of the mesh to faecal contamination.

Clinical examination as a means of primary diagnosis of parastomal hernias is subjective and is especially difficult in obese patients.^7,8^ The use of clincial examination and predetermined, defined endpoints is a weakness inherent in many of the current studies in this field.^4^ A study from 2011 demonstrated that clinical examination by experienced colorectal surgeons with a specialist interest in parastomal hernias had poor inter-observer reliability in identifying the presence or abscence of a parastomal hernia.^11^ A strength of our study is using a uniform, predetermined, blinded, objective method to identify the presence of a parastomal hernia. Defining strict inclusion and exclusion criteria allowed comparison of radiological outcomes between homogenous groups of patients undergoing surgery for the same indication by the same operating surgeons, using a uniform technique.

It has been acknowledged in the literature that clear outcome measures regarding incisional and parastomal hernias are lacking. Undoubtedly, further evaluation of parastomal hernias should involve both objective and radiological variables as well as clinical parameters in order to identify those hernias likely to cause symptoms and/or require repair and to assess the efficacy of interventions such as prophylactic mesh placement. Weaknesses of this study may include a lack of clinical/symptom correlation with the size and grade of parastomal hernias.

## Conclusions

Prophylactic mesh placement at the time of the index procedure reduces the diameter of abdominal wall aperture and the incidence of parastomal hernias containing bowel. A longer follow-up period and clinical correlation would be useful in assessing the long-term efficacy and safety of prophylactic mesh in preventing parastomal hernia development. Future studies should use both objective radiological as well as clinical endpoints when assessing parastomal hernia development with and without prophylactic mesh.
